# Polymorphic Cytochrome P450 Enzymes (CYPs) and Their Role in Personalized Therapy

**DOI:** 10.1371/journal.pone.0082562

**Published:** 2013-12-10

**Authors:** Sarah C. Preissner, Michael F. Hoffmann, Robert Preissner, Mathias Dunkel, Andreas Gewiess, Saskia Preissner

**Affiliations:** 1 Charité Universitätsmedizin Berlin, ECRC - Structural Bioinformatics Group, Deutsches Konsortium für Translationale Krebsforschung (DKTK), Berlin, Germany; 2 Institute for Laboratory Medicine, Berlin, Germany; 3 Charité Universitätsmedizin Berlin, Dental, Oral and Maxillary Medicine, CC3, Berlin, Germany; Southern Illinois University School of Medicine, United States of America

## Abstract

The cytochrome P450 (CYP) enzymes are major players in drug metabolism. More than 2,000 mutations have been described, and certain single nucleotide polymorphisms (SNPs) have been shown to have a large impact on CYP activity. Therefore, CYPs play an important role in inter-individual drug response and their genetic variability should be factored into personalized medicine. To identify the most relevant polymorphisms in human CYPs, a text mining approach was used. We investigated their frequencies in different ethnic groups, the number of drugs that are metabolized by each CYP, the impact of CYP SNPs, as well as CYP expression patterns in different tissues. The most important polymorphic CYPs were found to be 1A2, 2D6, 2C9 and 2C19. Thirty-four common allele variants in Caucasians led to altered enzyme activity. To compare the relevant Caucasian SNPs with those of other ethnicities a search in 1,000 individual genomes was undertaken. We found 199 non-synonymous SNPs with frequencies over one percent in the 1,000 genomes, many of them not described so far. With knowledge of frequent mutations and their impact on CYP activities, it may be possible to predict patient response to certain drugs, as well as adverse side effects. With improved availability of genotyping, our data may provide a resource for an understanding of the effects of specific SNPs in CYPs, enabling the selection of a more personalized treatment regimen.

## Introduction

Inter-individual variability of drug response and drug clearance is a complex and common problem in clinical practice [1]. Overlapping substrate specificity of enzymes, a multitude of single nucleotide polymorphisms (SNPs) [2] and variations between ethnic groups [3] make prediction of phenotypic drug response difficult. To avoid treatment failure and unnecessary toxicity, tailoring dosages and drug-cocktails for each individual is essential [4].

Differences in drug response can be attributed to variability in DNA sequences of specific genes which’s products are crucial for drug metabolism. For instance, SNPs in phase 1 enzymes, such as cytochrome P450 oxidases (CYPs) [3], phase 2 enzymes, such as Uridine 5'-diphospho-glucuronosyltransferase (UGTs) [5], and absorptive and efflux transporters, such as ATP-binding cassette transporters (ABC-transporters) [4], have been previously reported. Characterization of these enzymes and the effects of minor allele variants on the metabolism of specific drugs have been described in the literature and have recently been compiled by our group into a comprehensive database called SuperCYP [6]. Phase I reactions include oxidation, reduction, hydrolysis and cyclization. Using oxygen and NADPH as a co-substrate, CYPs are the major enzymes responsible for catalyzing such reactions [7] and account for approximately 75% of total drug metabolism [8]. 

The Human Genome Project identified 57 human CYPs, which were classified into 18 families and 43 subfamilies based on sequence similarity [9]. CYP families 1, 2 and 3 are responsible for metabolism of drugs, xenobiotics and certain endogenous molecules [3] and hence are of particular relevance to this current study. Most CYPs metabolize more than one drug. Similarly, a drug is often metabolized by multiple CYPs. Drugs can also inhibit or induce CYP activity, either by directly interacting with the enzyme or altering its expression. Characterization of these interactions is important to determine and predict compatible drug combinations [10]. Human CYPs are primarily membrane-associated proteins [11] that are ubiquitously expressed in most tissues. Highest expressions are generally found in liver tissue, but the distribution of particular CYPs varies [12], which indicates that the actual efficiency of a drug is likely to depend on CYP expression in the target tissue. There are significant inter-individual differences in enzyme activity leading to distinct phenotypes. For example the most frequent phenotype of CYP 2D6 is the extensive-metabolizer (78.8%), followed by intermediate- (12.1%), poor (7.6%) and ultra-rapid metabolizers (1.5%) [13]. 

In addition to drug catabolism, many CYPs are responsible for activation of prodrugs, such as cancer therapeutics [14] and antipsychotics [15]. Prodrugs are pharmacologically inactive compounds that require activation via metabolic conversion [16], allowing control of where, when and how much drug activity occurs [17]. This is particularly important for chemotherapeutic drugs, where the active drug ideally only acts on tumor cells in order to reduce toxic side effects [18]. Prodrugs can be activated by photo irradiation [19], change in pH [20] or enzymatically [21], for instance by CYPs [22]. Polymorphisms in CYPs can result in ineffective or aberrant activation of prodrugs [22], which can lead to toxicity [4]. Fortunately, advances in genetic research have made genotyping of a large number of patients possible, leading to identification of SNPs that alter expression or activity of drug metabolizing enzymes [3]. In this study we set out to determine the most frequent CYP polymorphisms having the highest impact on drug metabolism in Caucasians. This knowledge could facilitate the development of tests for efficient genotyping of patients thus leading to a better and more personalized treatment. 

## Methods

### Text mining

**Figure 1:**
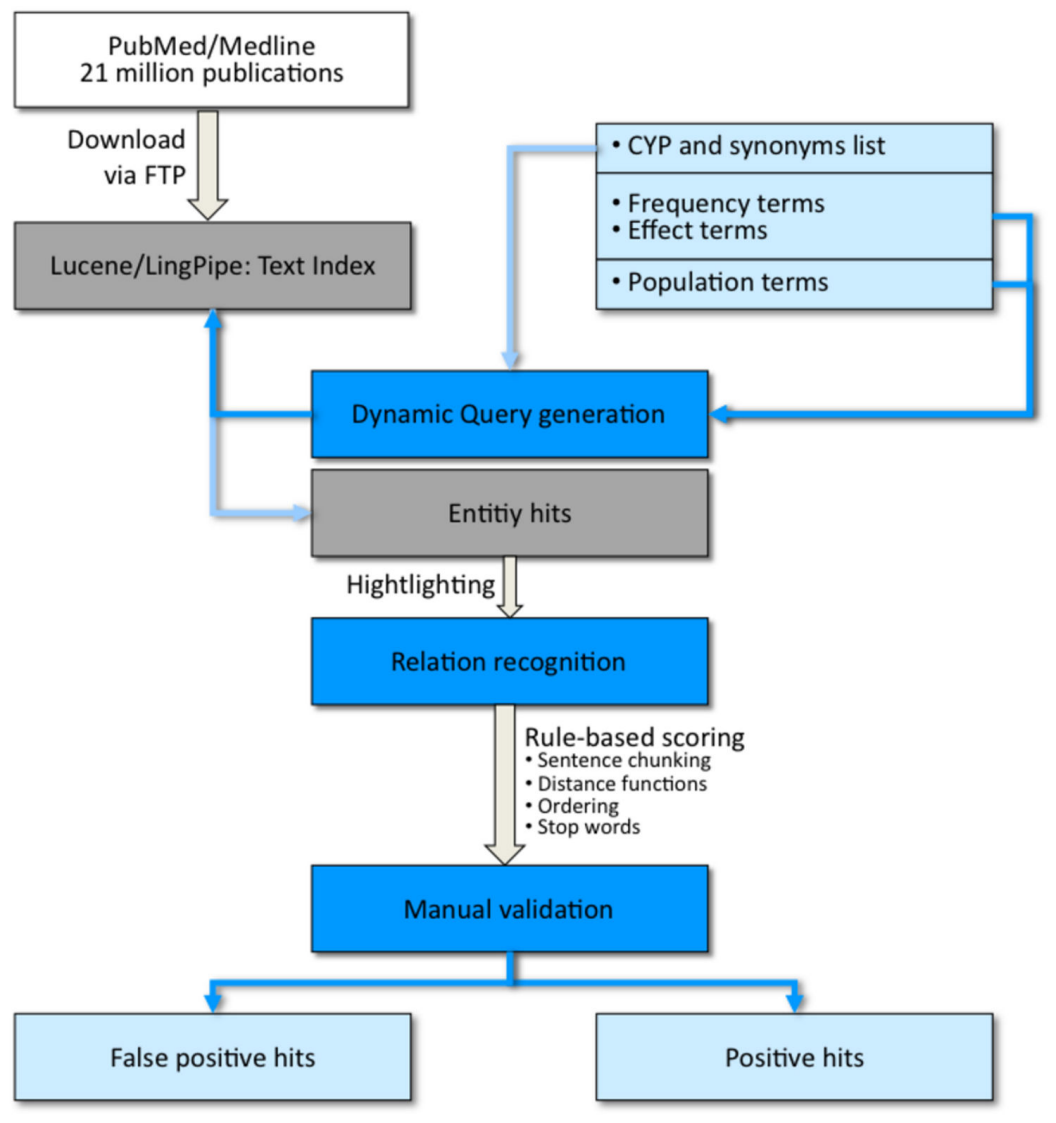
Flow-chart of the methods used for the text mining approach.

Information on drug metabolism can be found in more than 100,000 PubMed articles, yet limited data is available regarding the frequencies of SNPs in human CYPs. To identify relevant articles, a specific search tool was developed for text mining literature using Apache Lucene™ (http://lucenenet.apache.org) as a search engine library and LingPipe (http://alias-i.com/lingpipe). Figure 1 summarizes the different methods used for the textmining approach. Complete Medline/PubMed data were downloaded from the NCBI FTP site in xml-format and then indexed. The indexed data was dynamically queried by a search engine written in Java that outputs an sql‑file with the text mining hits, which served afterwards for manual validation. The search engine comprises several lists of synonyms for identifying entities, such as chemical compounds, biological targets, genes, cell types and polymorphisms, as well as interaction-related entities. If available, information on CYP polymorphism was extracted from the literature. Definitions and synonyms are included from UMLS® Metathesaurus®, that contains millions of biomedical and health related concepts, their synonymous names, and their relationships. As an example, the query for CYP2C19 was like: 

(Abstract: CYP2C19\** OR Title: CYP2C19\**) AND (Abstract: population OR Title: population) AND (Abstract: effect OR Title: effect) AND (Abstract: frequenc* OR Title: frequenc*). 

**Figure 2:**
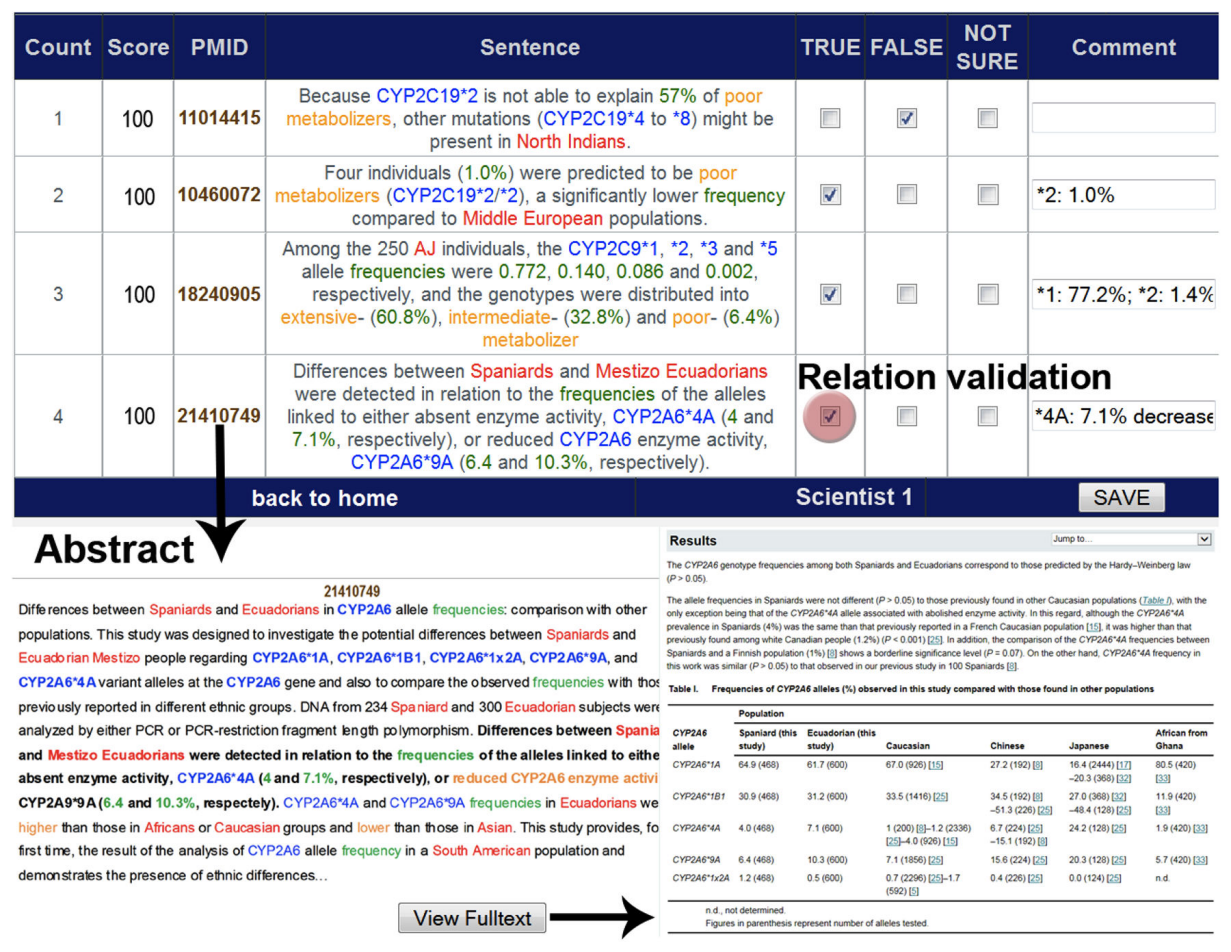
Text mining validation tool. The table shows the text mining validation tool with columns for score, PubMedID, relation sentence and checkboxes for the validation. The SNPs are highlighted in blue, frequencies in green, effects in orange and the ethnicities in red. ‘Scientist 1’ reads the abstract and, if necessary, has access to full text. Afterwards, the relation has to be validated as ‘true’, ‘false’ or ‘not sure’. If the relation is ‘true’, the relation is copied into the ‘comment’ field. These relations are copied into a new sql-file. If ‘Scientist 1’ activates the ‘not sure’ field, the relation has to be validated again by another scientist.

The term ‘CYP2C19’ was replaced through each human CYP and synonyms, as well as different ethnicity and outcome terms were used for ‘population’ and ‘effect’. The positional distance between the different terms had to be restricted to reduce false positive hits, when terms occurred far from each other in the abstract. The records found were scored rule-based. The rules employed order, redundancy, distance, topic segmentation and sentence breaking for boundaries. For example, a distance ≦ 7 between the CYP and the ethnicity and ≦ 6 between the frequency and the CYP was given a score of 100. Greater distances and negative interaction words resulted in lower scores. Duplicates were removed and a team of scientists manually processed 1,037 papers found in PubMed for relevance to polymorphisms and their frequency in Caucasian populations. The team consisted of three medical scientists, with three years experience in validation of text mining results. During this time, they reviewed over 10,000 abstracts with the focus on CYPs. A weekly meeting took place to ensure and raise the quality of text mining and to discuss problems. The aim was to achieve a coherent review operation. The text mining validation tool is shown in Figure 2. CYP polymorphisms that occurred with a frequency of more than one percent in the Caucasian population were included in this study.

### Localization of SNPs in a 3D CYP model

**Figure 3:**
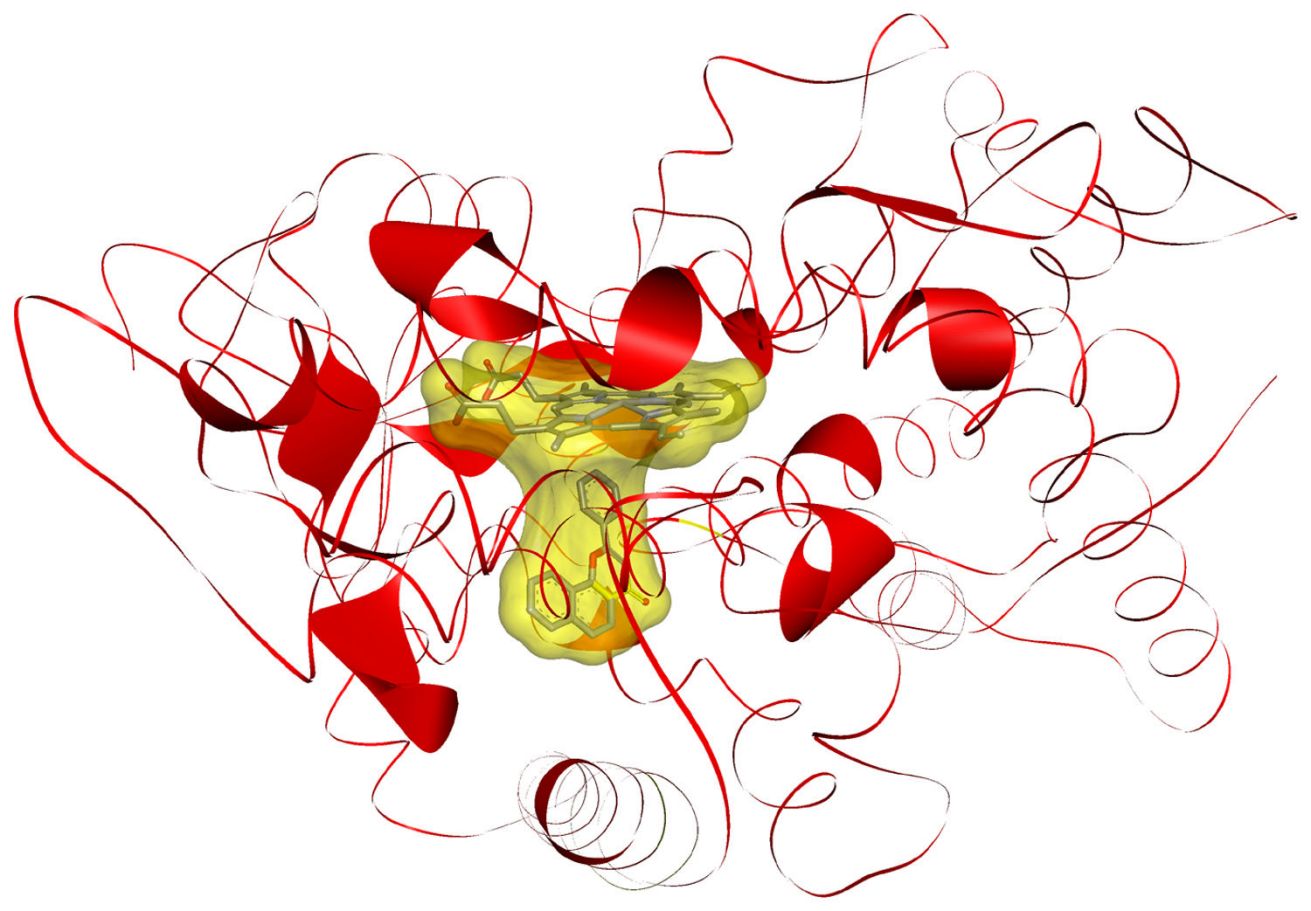
3D structure of a cytochrome with heme and a ligand with localization of frequent SNPs. Frequent SNPs in the four most polymorphic CYPs (1A2, 2C9, 2C19 and 2D6) were labeled in this 3D model of CYP 2D6 (PDB ID: 3TDA). Therefore, the ribbon was enlarged in the appropriate positions. The binding-side (transparent, orange colored surface) contains an iron ion and a porphyrine ring (heme). Frequent mutations of CYPs at the binding-side occurred at the following positions: 67, 89, 107, 117, 118, 120, 125, 132, 151, 201, 227, 261, 325, 377, 382, 386, 410, 454, 456, 469 and 470.

The evolutionary conservation taken from a multiple sequence alignment of CYPs was projected onto the 3D structure using CYP 2D6 as template (PDB ID: 3TDA). Frequent SNPs in the four most polymorphic CYPs (1A2, 2C9, 2C19 and 2D6) were labeled in the 3D model. The number of mutations was used to determine the thickness of the ribbon (Figure 3).

### CYP SNPs and 1,000 Genomes

The 1,000 Genomes Project (www.1000genomes.org) is an international initiative designed to provide full genomic sequence information from an ethnically diverse population [23]. CYP SNPs in 1,092 individuals were extracted using the online data slicer from the 1,000 Genomes Project (http://browser.1000genomes.org). Frequency analysis focused on non-synonymous coding SNPs with a prevalence of one percent or higher in all genomes regardless of ethnicity. The search included the main 29 CYP alleles from “The Human Cytochrome P450 (CYP) Allele Nomenclature Database” (http://www.cypalleles.ki.se/) [24]. In addition, 16 CYP alleles not listed in the CYP allele database due to very heterogeneous distributions were included. The 1,000 Genomes Database includes SNP effect predictions on CYPs, calculated by PolyPhen [25], which predicts possible functional alterations in human proteins after amino acid substitution based on physical and comparative considerations [26]. 

### Expression data

Affymetrix data was used to compare human CYP mRNA expression in 41 different types of tissue, further subcategorized into different regions of an organ, yielding a total of 65 tissue types. The series of datasets obtained from GEO (Gene Expression Omnibus, http://www.ncbi.nlm.nih.gov/geo/) were originally generated from 10 post-mortem donors (5 females and 5 males), and represent normal human tissues (Series GSE3526) [27]. The 84 probe sets, which measure the expression level of CYPs were normalized and assigned to 40 types of CYPs. To display differences in expression, a heat-map was generated using Genesis software [28]. Relative expression was calculated as the intensity of the gene in the region minus the mean intensity of the gene in all regions then divided by the standard deviation. This heat-map served as data source for the CYP body map in which only two-fold decreased or increased values were considered.

Our work would not have been possible without the publicly available datasets mentioned above. We are grateful and honor the work of involved research groups. 

## Results

### Frequencies of SNPs in CYPs

**Figure 4:**
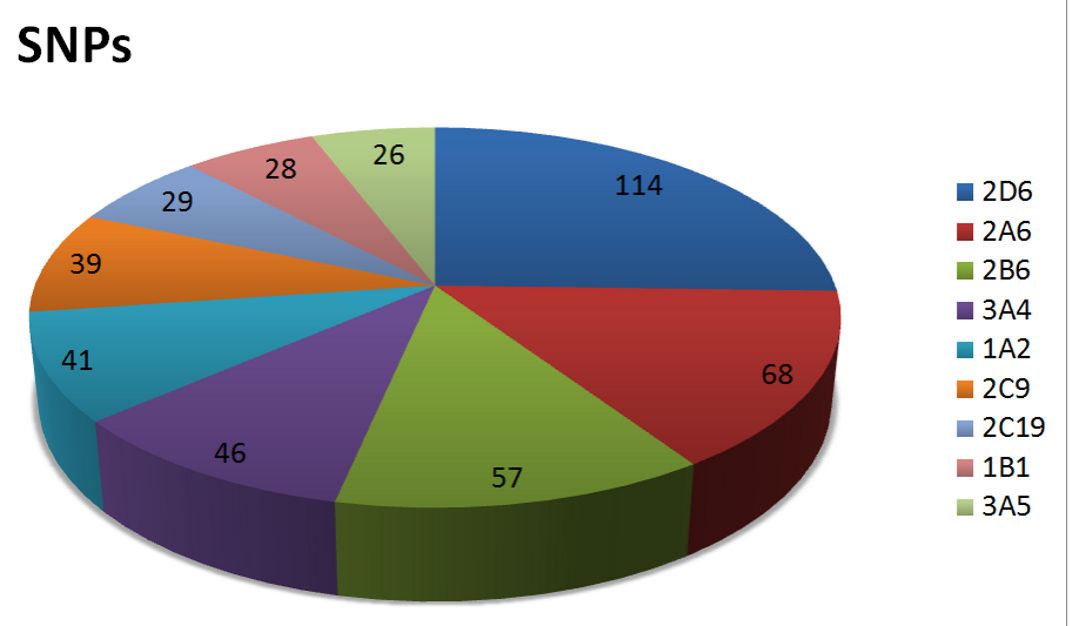
Number of known SNPs per CYP. The pie chart illustrates the number of known SNPs in different CYPs.

Analysis of the SNPs identified by text mining, showed that SNPs predominantly occurred in 3 polymorphic CYPs (2D6, 2A6 and 2B6) regardless of ethnic group. Only frequencies of known nucleotide changes were assessed to identify the extent of SNPs in CYPs. Figure 4 displays 9 CYPs, including 2D6 (114 SNPs), 2A6 (68 SNPs) and 2B6 (57 SNPs), which showed the highest number of SNPs. For other CYPs, the number of known SNPs was less than 22. CYP 2D6 is a major polymorphic CYP and, as expected, was the greatest contributor of polymorphic alleles in Caucasians.

### Allelic frequency in CYPs of Caucasians

The PubMed search yielded articles on 34 different CYP alleles with an occurrence greater than one percent in the Caucasian population (Table 1), which may be indicative of altered substrate metabolism. CYP 2D6 and 2B6 possessed the largest number of alleles with a known impact on metabolism in Caucasian population (11 and 6, respectively). Maximum allele frequencies in CYP 2D6 varied from 20.7% to 32.4%. When considering all 34 alleles, the most frequent alleles were CYP 3A5*3C at 81.3% (decreased enzyme activity) followed by CYP 1A2*1F at 33.3% (increased enzyme activity). Furthermore, the major alleles leading to increased metabolism were CYP 2A6*1B (30.0%), 3A4*1B (17.0%), 1A1*2A (19.0%) and 2C19*17 (18.0%). In contrast, decreased metabolism was attributed to 2D6*2A (32.4%), 2D6*4 (20.7%) and 2C9*2 (16.0%). Carrying the 2A6*4 allele (1.0%) leads to an inactive enzyme with no detectable substrate metabolism.

**Table 1:** CYP SNP frequencies in Caucasians.

**CYP**	**Allele**	**Amino Acid**	**Caucasian (%)**	**Enzyme Activity**	**Test Drug**	**References (PMID)**
**1A1**	*2A	I462V	19.0	increase	17β-estradiol	19514967
**1A2**	*1F	none	33.3	higher inducibility	Omeprazole	12534642 / 22299824
	*1D	none	4.82	decrease	Clozapine	12534642 / 20797314
**2C9**	*2	R144C	19.0% *1/*2 1.6% *2/*2 1.8 % *2/*3	decrease	Warfarin	15284536
	*3	I359L	9.0	decrease	Tolbutamide	11678789
**2C19**	*2	Splicing I331V defect	16.0	decrease	Clopidogrel	10460072
	*17	I331V	18.0	increase	Omeprazole	21247447
**2D6**	*3	N166D; 259 Frameshift	2.04	decrease	Debrisoquine	9012401
	*4	P34S; L91M; H94R; Splicing defect; S486T	20.7	decrease	Dextromethorphan	9012401
	*4D	P34S; Splicing defect; S486T	3.4	decrease	Bufuralol	11266079
	*4L	P34S; Splicing defect; S486T	4.5	decrease	Bufuralol	11266079
	*5	CYP2D6 deleted	4.1	no enzyme		9511177
	*6	118Frameshift	1.3	nonfunctional		9511177
	*7	H324P	1.0	decrease	Sparteine	9089660
	*9	K281del	2.0	decrease	Sparteine	9511177
	*10	P34S; S486T	8.0	decrease	Metoprolol	9511177 / 11505219
	*41	R296C; Splicing defect; S486T	8.0	decrease (expression)		15289790
**2A6**	*12	10 aa substitutions	2.9	decreased (expression)		16041240
	*1B	none	32.6	increase	Caffeine	22850738
	*2	L160H	2.3	decrease	Nicotine	11259354
	*4	CYP2A6 deleted	1.0	no enzyme		11259354
	*9	(TATA box)	7.1	decreased	Nicotine	15475735
**3A4**	*17	F189S	2.0	decrease	Testosterone	11714865
	*1B	none	17.0	increase (transcription)	Tacrolimus	12692107
	*2	S222P	2.7	decrease	Nifedipine	10668853
**3A5**	*3C	Splicing defect	81.3	decrease	Sirolimus	17162466
	*3k / *10	Splicing defect; F446S	2.0	decrease	Nifedipine	12893984
**3A7**	*2	T409R	8.0	increase	Dehydroepiandrosterone	15903124
**2B6**	*2	R22C	5.3	increase	Artemether	21746968 / 12242601
	*5	R487C	14.0	decrease	Nirvanol	11470993
	*4	K262R	5.0	increase	Bupropion	14515060
	*6	Q172H; K262R	25.2	decrease (expression)	Cyclophosphamide	14515060
	*7	Q172H; K262R; R487C	3.0	decrease	7-ethoxy-4-trifluoromethylcoumarin	12242601 / 14551287
	*22	none	3.0	increase (transcription)		15722458

Polymorphisms that are relevant for Caucasians are shown here with CYP, allele, amino acid and frequencies and their effect on enzyme activity. The test drug and the PubMed ID complete the table. A more detailed table including gene information and frequencies in other ethnic groups can be found in Table S1.

Comparison among different ethnic groups revealed that frequencies differed considerably and displayed a heterogeneous distribution of CYP alleles. For instance, in Asian and African populations, CYP2A6*2 possessed a frequency of 28.0% and 62.0%, respectively, whereas a frequency of 8.0% was observed in Caucasians. A more detailed table with additional information on CYP SNPs in Caucasians and other ethnic groups is available in Table S1. 

### Major drug metabolizing CYPs

**Figure 5:**
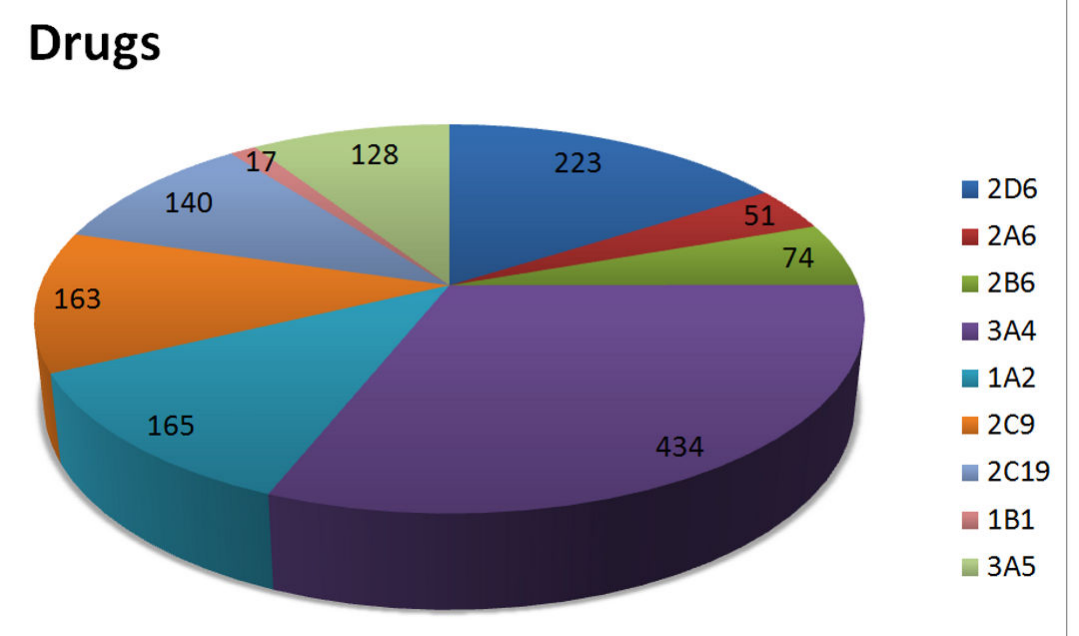
Number of drugs metabolized per CYP. The pie chart illustrates the number of drugs that can be metabolized by a specific CYP.

Not all 57 human CYPs are involved in drug metabolism. The primary CYPs responsible for drug metabolism were determined by first ranking the CYPs according to the total number of drug substrates (Figure 5). Twelve CYPs accounted for 93.0% of drug metabolism, regarding to the entire number of 1.839 known drug-metabolizing-reactions in the SuperCYP database. CYP 1A2, 2D6, 2C9 and 2C19 were responsible for nearly 40.0% of drug metabolism and including CYP 3A4 even for 60.0%. 

Since the described four CYPs are highly polymorphic and commonly occur in Caucasians, further detailed analyses were restricted to these four CYPs. On the overall CYP system, it is expected that these four CYPs would have the greatest impact on inter-individual variability of drug response. Although CYP 2A6 and 2B6 possess various relevant alleles in Caucasians, they do not cover a large range of drug interactions (51 and 74 substrates, respectively). 

All SNPs in the four most polymorphic CYPs (1A2, 2C9, 2C19 and 2D6) influenced enzymatic activity due to localization in the substrate-binding cavity as shown in Figure 3.

### Expression data

**Figure 6:**
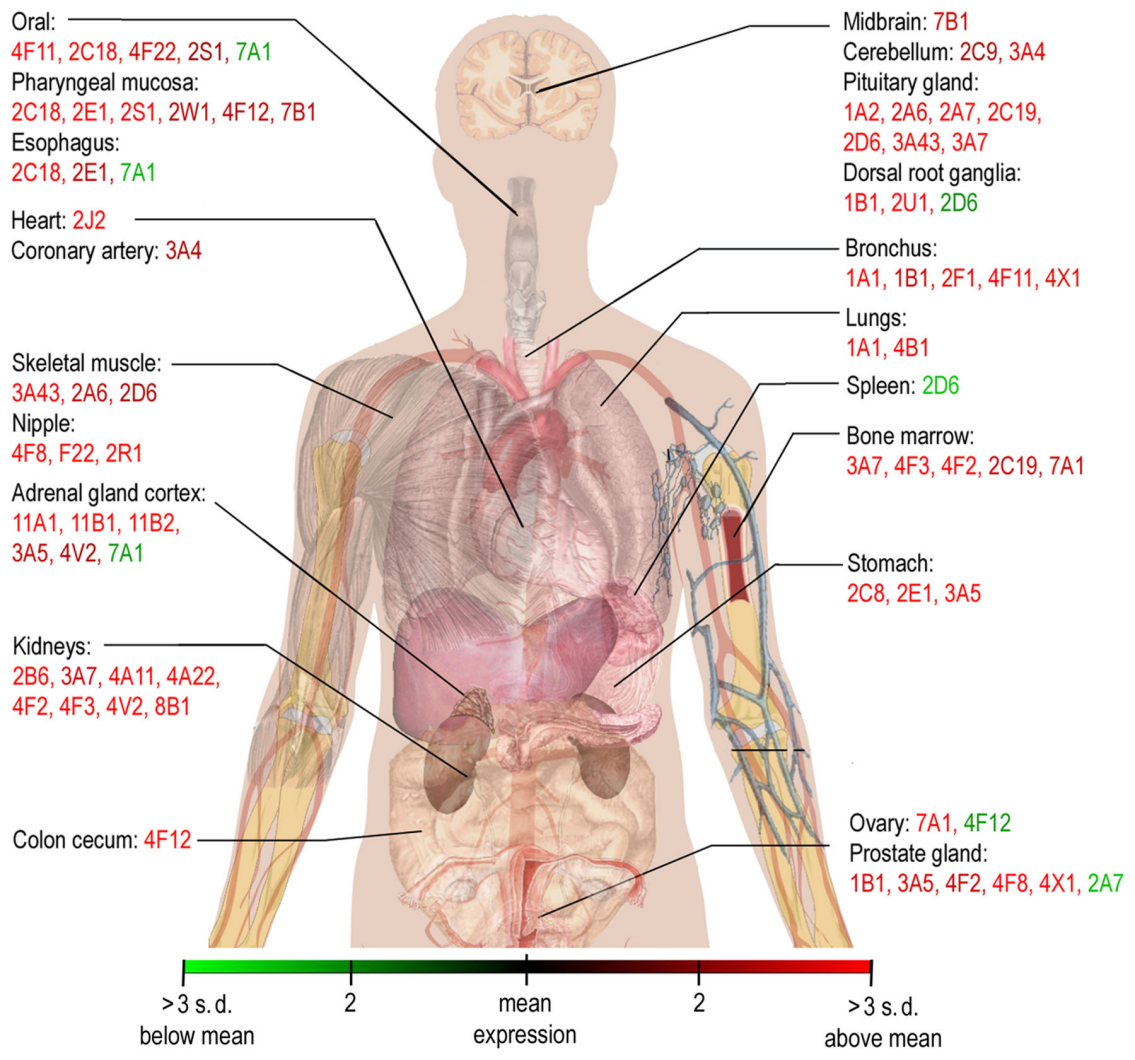
Body map of cytochrome P450 enzyme expression. A schematic map of the specific expression of different CYPs in human organs is presented. Expression values are relative to the mean expression in all organs. At least three-fold higher expression of a CYP in one organ is indicated by red text. At least three-fold lower expression is indicated by green text. A color spectrum for the expression values is illustrated in the provided scale. CYPs with average expression in one organ were not included.

Because of high CYP expression levels in some tissues, an impact of CYP isoforms in particular tissues can be deduced. The work of Nishimura and colleagues demonstrated differences in CYP mRNA expression in various human tissues. For example, CYP 2F1, 4B1, 4F8, 11S, 11A, 11B1, 11B2, 19 and 24 are not expressed in the liver [29]. The current study confirmed these results and extended the findings, which are shown in Figure 6. Nishimura analyzed mRNA levels of 30 CYP isoforms in 11 tissue types. Similarly, the current study investigated the expression of 40 CYP isoforms in 41 tissue types. The liver was considered separately in the analysis in order to identify the differences between the other tissues. In 21 different tissues, a heterogeneous distribution of CYPs was observed. For instance, 39 different CYP isoforms showed higher mRNA expression in at least one or more tissue types. Significant differences were observed in the adrenal gland cortex, which possessed 6-fold higher expression of CYP 11A1, 11B1 and 11B2 (compared to the mean expression). Interestingly, no other tissue showed high levels of expression of these three CYPs. Large differential expression compared to other tissues was also observed in the kidneys, where a 6-fold increase in CYP 4A22, a 5-fold increase in CYP 8B1 and 4-fold increases in 4V2, 4F2, 4A11 and 2B6 were noted. In addition, 5-fold higher expression of CYP 2C8 was found in lung, CYP 4F8 in prostate, 4F3 in bone, 2F1 in bronchial tubes and 2C8 in stomach. Furthermore, CYP 2C18 showed a high level distribution restricted to the oral cavity, pharynx and esophagus. Two-fold lower expression was detected for CYP 2A1 in the esophagus, 2A7 in the prostate, as well as 2C9 and 2D6 in the spleen. 

### CYP SNPs and 1,000 Genomes

The current study identified 199 non-synonymous coding SNPs with frequencies greater than one percent (Table S2). Compared to the “Human Cytochrome P450 Allele Nomenclature Database” (http://www.cypalleles.ki.se/), we found several SNPs in 1,000 Genomes not related to alleles defined and named in the Database. To elucidate the difference between the ‘The Human Cytochrome P450 Allele Nomenclature’ and 1000genome data regarding new SNPs, we examined CYP2A6 exemplary. Table 2 summarizes SNPs most likely to alter enzyme activity [25]. It displays five SNPs, which can lead to an altered enzyme activity with frequencies between 1.4 and 5.1 %. Only I471T (rs5031016) is also contained in the CYP nomenclature and reflects the CYP2A6*36 allele. New updates have to be done to map a comprehensive CYP SNP data source. 

**Table 2:** Differentially expressed CYPs and their SNP frequencies.

**CYP**	**ID**	**Mutation**	**Amino Acid**	**Global frequency (%)**	**CYP**	**ID**	**Mutation**	**Amino acid**	**Global frequency (%)**
3A43	rs45450092	435G>T	M145I	1.5	2F1	rs144315434	1172T>C	L391P	5.4
	rs45621431	825G>A	M275I	2.3		rs146029724	1330A>C	M444L	6.5
	rs680055	1018C>G	P340A	13.4	2W1	rs61746347	557G>A	R186H	2.8
	rs78548296	389G>A	R130Q	1.0		rs117826462	547C>G	L183V	1.4
4A11	rs1126743	1374C>G	I458M	42.6	4F11	rs1060463	1271G>A	R424Q	49.5
	rs4926581	553G>T	V185F	28.1		rs148197835	538C>T	R180C	4.2
	rs61736429	1525C>T	L509F	2.1		rs57519667	436C>T	R146C	1.6
	rs62618709	553G>T	V185F	1.1	2C18	rs115091705	431G>A	R144H	1.7
4A22	rs112604161	181G>A	G61R	1.6		rs117111102	370C>T	R124W	1.4
	rs113777592	553G>T	V185F	29.6		rs2281891	1154C>T	T385M	19.3
	rs2056900	388G>A	G130S	29.9		rs41286880	1004G>A	R335Q	2.5
	rs4926600	1525C>T	L509F	12.9		rs79500998	1324C>T	R442C	1.0
	rs61507155	311A>T	Y104F	6.6	7A1	rs8192875	1039G>A	D347N	1.6
	rs61736431	1154C>T	P385L	1.2	4F12	rs16995378	47C>T	T16M	7.6
4B1	rs12094024	986A>C	Y329S	2.2		rs57578760	808G>C	V270L	3.7
	rs2297809	1123C>T	R375C	18.3		rs76142062	88C>A	L30I	3.4
	rs4646487	517C>T	R173W	16.8	11B1	rs11775687	562C>T	P188S	5.7
	rs59694031	1109G>C	C370S	4.0		rs9657020	593C>T	T198M	12.7
4F2	rs2074900	515C>T	Thr172I	25.4	4F3	rs118159249	1420G>A	A474T	1.1
	rs2108622	1297G>A	V433M	20.9	2A7	rs111390860	988C>T	R330W	1.0
	rs3093153	554G>T	G185V	3.7		rs184466431	1301G>T	R434L	1.2
	rs3093200	1555C>A	L519M	8.4		rs3869579	778C>T	R260C	46.7
5A1	rs13306050	1372C>T	R458C	3.3		rs60711313	1259T>C	I420T	3.2
	rs13306052	679GA	V227M	1.4		rs75152309	1106A>T	K369M	6.6
	rs6952940	544C>T	P182S	2.4		rs78754793	244G>C	A82P	2.4
2A6	rs5031017	1436G>T	G479V	1.4	2S1	rs34971233	1397C>T	P466L	1.1
	rs5031016	1412T>C	I471T	5.1	2E1	rs6413419	535G>A	V179I	7.2
	rs28399499	983T>C	I328T	2.3		rs28969387	1370A>T	H457L	6.3
	rs8192709	64C>T	R22C	4.5	2C19	rs17884712	431G>A	R144H	1.4
	rs28399499	383T>C	I128T	2.3	8A1	rs5626	706C>T	R236C	3.7
2C8	rs11572103	805A>T	I269F	16.4	2D6	rs2982054	986G>A	R329H	31.2
	rs1058930	792C>G	I264M	4.1		rs1058172	941G>A	R314H	7.9
	rs11572103	805A>T	I269F	16.4		rs59421388	859G>A	V287M	5.3
2C9	rs28371686	1080C>G	D360E	2.3		rs1065852	100C>T	P34S	25.9
	rs28371685	1003C>T	R335W	2.0	1A1	rs4646422	134GA	G45D	6.7
	rs2256871	752A>G	H251R	4.0		rs17861094	233T>C	I78Thr	8.3

Data was extracted from the 1,000 Genomes Project site (http://www.1000genomes.org/) [23]. A more detailed table can be found in the supplemental material. Possibly CYP-activity damaging SNPs are included in Table S2.

With the potential to alter drug metabolism, the 72 listed SNPs occurred in 24 CYPs. The most frequent SNPs were CYP 4A11 rs112743 (42.6%; highly expressed in kidney tissue), CYP 4F11 rs1060463 (49.5%; highly expressed in bronchus tissue) and CYP 2A7 rs3869579 (46.7%; highly expressed in the pituitary gland but low in the prostate gland). Our study findings show that some CYPs are not only heterogeneously expressed, but also highly polymorphic. 

## Discussion

### Genetic diversity and polymorphisms

Mutations in a CYP gene can lead to functional alterations, such as increased or decreased activity. If a mutant allele occurs at a frequency of at least one percent in a population, it is referred to as a pharmacogenetic polymorphism. Such polymorphisms can be discovered at the genotype level and/or the phenotype level based on altered function of the enzyme [30]. 

Individuals in a population can be stratified according to metabolic ratios of particular CYPs, which have great clinical relevance. For example, a CYP 2D6 poor metabolizer should not be administered codeine since the drug would have no effect. Conversely, a CYP 2D6 ultra-rapid metabolizer would likely suffer side effects from a normal dosage [31,32]. CYP 2D6 is a highly polymorphic CYP with at least 70 allelic variants [33] that can be categorized into four phenotypic classes. Overall CYP 2D6 expression in liver tissue is only approximately 2%, but hundreds of drugs are metabolized by this enzyme, including opiates, beta-blockers, anti-arrhythmics, tricyclic antidepressants, SSRIs, 5-HT3-antagonists and neuroleptics [34]. About 10% of the Caucasian population have difficulties in fully metabolizing these drugs [35], leading to harmful side effects [32,36]. Therefore, personalized prescriptions will become of great importance [37].

### Personalized medicine

Since 2009, the Clinical Pharmacogenetics Implementation Consortium (CPIC) provides information on how genetic test results can be used to optimize drug therapy. The guidelines center on genes or on specific drugs. For some drugs, they also provide dosing guidelines for clinicians [38].

#### Psychiatric drugs

As most psychiatric drugs are metabolized by highly polymorphic CYP 2D6 and CYP 2C19, psychiatrists were first to propose the idea of CYP genotyping [39–41]. Three state hospitals in Kentucky recruited 4,532 psychiatric patients for genotyping of both CYPs with the help of DNA microarray technology. 

Results from the current study were consistent with previous studies of allele frequency [35], demonstrating the importance of personalized prescription given that more than one tenth of patients are not likely to respond to standard treatment and suffer unwarranted toxicity. In the study performed by de Leon and colleagues, the dosage was adapted to the guidelines of Kirchheiner [15] for antipsychotics and antidepressants. The authors propose a numeric dosage adaptation system that reflects expression of CYP 2D6 and CYP 2C19. 

#### Cardiovascular drugs

An important area of focus is stent implantation and/or inhibition of blood clots after an acute coronary syndrome (ACS) to prevent ischemic events. Therefore, antiplatelet agents are administered before and after percutaneous coronary intervention (PCI) to reduce the risk of ischemic events. Currently, the gold standard therapy is a combination of aspirin and clopidogrel [42,43]. Unfortunately, approximately 29% of people respond poorly to clopidogrel [44] and, therefore, have an increased risk for recurrent ischemic events after PCI [45]. Several different factors were discovered to contribute to the variability in clopidogrel response, including polymorphisms, impaired absorption or bioavailability, poor compliance and pre-existing conditions (increased body mass index, diabetes mellitus, ACS) [46]. In addition, clopidogrel is a prodrug that requires activation through the CYP system. The activated metabolite inhibits the ADP P2Y12 receptor [47]. Polymorphisms causing loss of function in the CYP system are associated with poor drug response. Most notably, the CYP 2C19*2 polymorphism was shown to lead to a 30% increased risk of major adverse cardiovascular events during treatment with clopidogrel [48–51]. Furthermore, the CYP 2B6*5 and P2Y12 polymorphisms are also associated with clopidogrel resistance [52]. In contrast, an enhanced response due to increased transcriptional activity occurs with the CYP 2C19*17 polymorphism, leading to increased risk of bleeding during clopidogrel therapy [53,54]. 

The CYP3A4*2 allele with a frequency of 2.7 % in Caucasian leads in vitro to reduced (six fold to nine fold) intrinsic clearance for nifedipine [55]. This could have a great influence on the tolerability of patients getting this dihydropyridine calcium channel blocker. Indications for nifedipine are widely distributed, e.g. Angina pectoris, Hypertonia, Achalasia and Raynaud's phenomenon, so the application is very common. An in vivo research regarding the alteration of nifedipine metabolism in CYP3A4*2 patients should be done, to possibly prevent toxic and/or increased side effects.

Previous findings described above, emphasize the importance of CYP polymorphisms and alternatively metabolized drugs in clinical practice. Prediction of CYP activity may be helpful to assess drug response. For instance, the (13)C-pantoprazole breath test, which measures CYP 2C19 activity, can detect clopidogrel resistance [56] and support use of suitable drug alternatives like Ticagrelor (no activation required, metabolized via CYP 3A4). 

### Additional observed effects

Apart from altered drug metabolism, CYP polymorphisms were also potentially associated with neoplastic growth, adverse psychological behavior and other diseases. In women, polymorphisms in CYP 1A1 seemed to increase susceptibility to genital cancers [57,58]. Conversely, the CYP 2D6*4 polymorphism has been shown to have a protective effect against breast cancer [59]. Furthermore, 2C19*2, 2D6*4, 2D6*10 and 1A1*2A have been associated with increased risk of head and neck squamous cell carcinoma [60]. 

In addition to the role that CYP polymorphisms play in pathological processes and susceptibility to certain diseases, recent genome-wide association studies (GWASs) have demonstrated an association between increased coffee consumption and SNPs rs2472297-T (located between CYP1A1 and CYP1A2) and rs6968865 (next to aryl hydrocarbon receptor) [61]. Huo et al. (2012) determined that certain SNPs are associated with increased susceptibility to schizophrenia [62], while Peñas-Lledó and colleagues found a positive association between the extent of active CYP 2D6 and frequency of suicide attempts, providing evidence that CYP diversity may need to be accounted for in clinical practice [63]. 

### Diversity of expression in human tissues

Variable expression of functionally distinct CYP isoforms across different tissue types indicates that certain isoforms play specific roles in a tissue-dependent manner. Figure 6 provides an illustrative overview of CYP expression in the human body. Such knowledge may be useful for development of new prodrugs activated by a specific CYP highly expressed in the preferentially targeted tissue, ultimately leading to increased bioavailability at the target site and reduced side effects. On the other hand, variable expression of CYPs in different tissues may adversely affect drug efficacy in some tissues. Such a case could occur if drugs undergo an inactivation through a higher expressed CYP in their target tissues. Regardless, further clinical investigation is required. 

Even polymorphic CYP isoforms show a heterogeneous tissue distribution. In particular, CYP 1A2, 2C19 and 2D6 are highly expressed in the pituitary gland. Furthermore, highest expression of CYP 2C9 was detected in the cerebellum, while greatest expression of 2D6 and 2B6 were found in skeletal muscle and kidneys. The influence of mutations in CYPs in particular organs remains to be determined and requires further investigation. 

Differential distribution of CYPs may have an influence on specific side effects of drugs. For example, cyclophosphamide (CPA) therapy can lead to development of hyponatremia. CPA is a prodrug converted by CYP 2B6 into the active form [64]. The hyponatremia is the result of increased expression of aquaporins 1 and 7, which is induced by CPA [65]. CYP 2B6 has high expression in kidneys, indicating that a higher level of active CPA is likely to occur in the kidneys and lead to the undesirable side effect. 

## Conclusions

In summary, the current study identified four major CYPs (1A2, 2D6, 2C9 and 2C19) and 34 polymorphic alleles with a significant impact on the drug metabolism in the Caucasian population. Once genomic testing becomes part of routine analysis, this data enables prediction of complications in drug therapy and development of a personalized treatment regimen, where drug dosages are based on an individual’s specific CYP profile [6]. Ultimately, this approach may prevent treatment failures and avoid unnecessary side effects. Another interesting field could be the consideration of CYP polymorphisms in clinical trials. Potentially, it would decrease the failures if information of potential polymorphisms in different ethnic groups was included. Findings from the current study will be included in the SuperCYP database. 

With the aim of assessing the effects of CYP polymorphisms on chemotherapy and establishing a cost efficient method to detect relevant CYP polymorphisms, a retrospective study in leukemia cells from pediatric patients is currently under way [66].

## Supporting Information

Table S1 **Extended table of CYP SNP frequencies in Caucasians and other ethnics.** The table includes the content of Table 1 and further information (other ethnics and nucleotide changes) that have been extracted by text mining.
(XLSX)

Table S2 **Extended table of differentially expressed CYPs and their SNP frequencies.** The extended table lists possibly CYP-activity damaging SNPs. CYP SNPs with frequencies (%) greater than one percent were included. The data was extracted from the 1,000 Genomes Project site (http://www.1000genomes.org/).
(XLSX)
